# Fatal Pulmonary Phaeohyphomycosis Associated with Large Vessel Thrombosis in a Dog

**DOI:** 10.3390/microorganisms14061219

**Published:** 2026-05-28

**Authors:** Clara Almeida Azerêdo, Nicole Sales de Almeida, Yasmim Couto e Coura, Juliana Mariotti Guerra, Nayara Bastos Costa, Bruna Samara Alves-Ribeiro, Adryanne Rezende Lobato, Alana Flávia Romani, Dirceu Guilherme de Souza Ramos, Klaus Casaro Saturnino

**Affiliations:** 1 Independent Researcher, Jataí 75800-257, Goias, Brazil; claraazeredo@gmail.com; 2 Residency Program in Veterinary Medicine, School of Veterinary Medicine and Animal Science (EVZ/UFG), Goiania 74691-835, Goias, Brazil; nicolesalesdealmeida@gmail.com; 3 Federal University of Viçosa, Viçosa 36570-900, Minas Gerais, Brazil; yasmim.coura@ufv.br; 4 Faculty of Veterinary Medicine and Animal Science, University of São Paulo, São Paulo 05508-900, São Paulo, Brazil; jumariotti.vet@gmail.com; 5 Laboratory of Veterinary Anatomical Pathology, Academic Unit of Agricultural Sciences, Federal University of Jataí, Jataí 75801-615, Goias, Brazil; nayara.costa@discente.ufj.edu.br (N.B.C.); brunasamara@discente.ufj.edu.br (B.S.A.-R.); adryannerezende@discente.ufj.edu.br (A.R.L.); 6 Federal University of Jataí, Jataí 75801-615, Goias, Brazil; alana_romani@ufj.edu.br (A.F.R.); dguilherme@ufj.edu.br (D.G.d.S.R.)

**Keywords:** dog, dematiaceous fungi, phaeohyphomycosis, pulmonary fungal infection, thrombosis, veterinary pathology

## Abstract

Phaeohyphomycosis is an opportunistic fungal infection caused by dematiaceous fungi and is considered uncommon in dogs, particularly when associated with visceral or systemic involvement. Pulmonary disease as a primary site of infection is rarely reported in veterinary medicine and is often associated with an unfavorable outcome. This report describes a fatal case of pulmonary phaeohyphomycosis in a dog, characterized by severe granulomatous pneumonia, vascular invasion by pigmented fungal hyphae, and the development of large vessel thrombosis. Histopathological examination revealed septate, pigmented hyphae consistent with dematiaceous fungi associated with an intense granulomatous inflammatory response. Although molecular analysis by polymerase chain reaction was unsuccessful due to the absence of amplifiable DNA in archived FFPE tissue, the clinicopathological correlation and histopathological findings were sufficient to support a diagnosis consistent with phaeohyphomycosis. Severe pulmonary inflammation likely contributed to vascular endothelial injury, resulting in pulmonary hypertension and thrombosis of major veins. This case highlights the diagnostic and clinical challenges associated with phaeohyphomycosis in dogs and emphasizes the importance of considering this infection in the differential diagnosis of chronic or progressive respiratory diseases accompanied by systemic complications. Furthermore, it reinforces the relevance of histopathology and comprehensive clinicopathological evaluation when molecular confirmation of the etiological agent is not achievable.

## 1. Introduction

The term phaeohyphomycosis refers to mycoses caused by pigmented, filamentous fungi belonging to the dematiaceous group, which contain melanin in their conidia, spores, and hyphae [1]. These fungi are considered opportunistic pathogens in animals and humans and are commonly found as saprophytes in warm and humid environments [2]. The disease has been associated with a wide range of clinical syndromes [3].

Although these fungi are cosmopolitan, phaeohyphomycosis is infrequently reported in both humans and animals [4]. The disease may present as cutaneous, subcutaneous, or systemic forms [5]. Diagnosis is primarily based on the histopathological identification of pigmented fungal structures associated with granulomatous inflammation; however, molecular techniques such as polymerase chain reaction (PCR) or immunohistochemistry are often required for species-level identification [1]. Nevertheless, fungal culture remains the gold standard for the etiological diagnosis of fungal infections. Although the pathogenesis of phaeohyphomycosis is not fully understood, melanin is believed to play a significant role in fungal virulence [6].

In humans, phaeohyphomycosis presents as a variety of clinical syndromes [3], whereas in animals, the disease has been more frequently reported in cats, typically causing cutaneous or subcutaneous lesions [2]. In dogs, reported manifestations include encephalitis [7], nephritis [1], systemic disease [4,8,9,10], cutaneous [11], subcutaneous [12], and osteolytic forms [13], with the systemic form being the most commonly described. Antoniassi [5] also reported a cutaneous presentation in a horse.

Clinical signs vary according to the organs affected but commonly include progressive weight loss, hyporexia, abdominal distension, dyspnea, vomiting, and lethargy [4]. Neurological manifestations [1] and cutaneous alterations have also been reported [11,12].

Similar to what is observed in humans, the prognosis of canine phaeohyphomycosis is guarded to poor. However, early treatment with newer triazole antifungal agents, such as voriconazole and posaconazole, in immunocompetent animals may result in favorable clinical outcomes. Localized disease may be amenable to surgical resection [14]; however, surgical feasibility depends on the location and extent of the lesions and may not be curative, with a high risk of recurrence and a lack of clear scientific consensus regarding optimal management [2].

Phaeohyphomycosis comprises a group of infections caused by dematiaceous fungi characterized by the presence of melanin in the fungal cell wall, capable of affecting different animal species and humans. Although these agents are widely distributed in the environment, infections in dogs are considered uncommon and are most often described as localized cutaneous or subcutaneous lesions. Reports involving systemic or visceral involvement, particularly cases with primary pulmonary infection and associated vascular complications, remain scarce in the veterinary literature. In this context, the present study aims to report a fatal case of phaeohyphomycosis in a dog, characterized by a probable pulmonary origin of infection, lack of molecular confirmation of the etiological agent, and development of large vessel thrombosis, highlighting the diagnostic challenges, clinical implications, and the importance of clinicopathological correlation in the recognition of this disease.

## 2. Material and Methods

A mixed-breed male dog, non-neutered, eight years old and weighing 15.85 kg, was admitted to a veterinary teaching hospital with a primary complaint of pain in the abdominal and thoracolumbar regions, in addition to progressive weight loss and softened greenish-colored feces. According to the owner, the patient exhibited increased water intake and increased urinary frequency. The animal had previous contact with rural environments during its early life but had been living in an urban area for several years, with outdoor access limited to supervised walks.

On physical examination, the patient was depressed, with a heart rate of 144 beats per minute (bpm). Cardiac auscultation revealed normorhythmic and hypophonetic heart sounds, and systolic arterial blood pressure was measured at 100 mmHg. The respiratory rate was 44 breaths per minute, mucous membranes were pale, and body temperature was 36.6 °C. Capillary refill time was prolonged (four seconds), skin turgor was decreased, and dehydration of approximately 8% was estimated. Reactive submandibular and popliteal lymph nodes were noted, and the body condition score was consistent with cachexia [15]. Signs of pain were observed during abdominal and thoracolumbar palpation. Alopecic areas with marked scaling were also identified on the skin.

Based on the anamnesis, clinical history, and physical examination findings, acute kidney injury, chronic kidney disease, and hemoparasitosis were initially suspected. Therefore, laboratory tests including a complete blood count, serum biochemical profile, urinalysis, urine protein-to-creatinine ratio, and abdominal ultrasonography were requested to assess the patient’s general condition and assist in establishing a definitive diagnosis.

The erythrogram revealed microcytic hypochromic anemia, with a red blood cell count of 3.51 × 10^6^/µL (reference range: 5.5–8.5 × 10^6^/µL), mean corpuscular volume (MCV) of 51.3 fL (60–77 fL), and mean corpuscular hemoglobin concentration (MCHC) of 31.7 g/dL (32–36 g/dL). Leukopenia was observed in the leukogram due to neutropenia (360/µL; reference range: 3000–11,500/µL), eosinopenia (0/µL; 150–1250/µL), and lymphopenia (229/µL; 1000–4800/µL). Serum biochemistry revealed creatinine, urea, and alanine aminotransferase (ALT) values within reference ranges; however, alkaline phosphatase (ALP) activity was increased, with a value of 361 IU/L (reference range: 20–156 IU/L) [16].

Urinalysis was performed using a cystocentesis-collected sample and revealed urine specific gravity within the reference range (1.032; reference range: 1.015–1.045), dark yellow coloration, pH of 6.0 (5.0–7.0), and moderate to marked proteinuria (100 mg/dL; reference: absent). Urinary sediment examination revealed a moderate number of transitional epithelial cells (++), a mild number of renal epithelial cells (+), a mild presence of granular casts (+), and a moderate amount of amorphous phosphate crystals (++). In the urine protein-to-creatinine ratio (UPC), urinary protein concentration was 212 mg/dL and urinary creatinine concentration was 50 mg/dL, resulting in a UPC value of 4.2. Based on these findings, significant proteinuria was diagnosed [17].

Due to the patient’s clinical condition, hospitalization was initiated for hemodynamic stabilization. Intravenous fluid therapy was administered using lactated Ringer’s solution at an infusion rate of 7 mL/kg/h during the first four hours, followed by 4 mL/kg/h for maintenance. The prescribed medications included Hyplex B^®^ (Hypofarma, São Paulo, SP, Brazil, 0.2 mL/kg, IV) and Bionew^®^ (Louveira, SP, Brazil, 0.2 mL/kg, IV) diluted in the fluid therapy, ondansetron (0.5 mg/kg, IV), dipyrone (25 mg/kg, IV), maropitant citrate (0.1 mL/kg, IV, administered slowly), omeprazole (20 mg, orally, one tablet), and Cobavital^®^ (Abbott of Brazil, Rio de Janeiro, RJ, Brazil, 4 mg/kg, orally).

After 18 h of hospitalization, the patient developed progressive abdominal distension, and an abdominal focused assessment with sonography for trauma (A-FAST) examination was performed. This examination revealed a small amount of gaseous content in the stomach and small intestine, as well as changes compatible with glomerulonephritis. After approximately 20 h of hospitalization, the patient exhibited marked abdominal distension, tachypnea (60 breaths per minute), tachycardia (180 bpm), and weak pulse. Abdominal radiography was performed and revealed a large amount of gaseous content in the stomach and intestinal loops.

Given the patient’s critical condition, nasogastric tube placement was initially attempted to decompress the stomach; however, due to unsuccessful placement, a gastrocentesis was performed using a needle (16G × 1 1/2″). The puncture was carried out in the left flank region, below the last ribs of the costal arch [18], allowing drainage of approximately 800 mL of yellowish, foamy liquid content. Complete drainage was not achieved, and exploratory laparotomy was considered. However, the patient’s clinical condition rapidly deteriorated, progressing to cardiorespiratory arrest. Eight cycles of cardiopulmonary resuscitation were performed [19], without success, and the patient died approximately 24 h after hospitalization.

The carcass was submitted for routine necropsy at the Veterinary Pathology Laboratory of the Federal University of Jataí (LAPVET-UFJ). Tissue samples were collected, fixed in 10% buffered formalin for 24 h, and routinely processed through graded ethanol dehydration, xylene clearing, and paraffin embedding. Sections of 5 µm thickness were obtained and stained with hematoxylin and eosin (H&E). During manuscript revision, residual archived pulmonary paraffin blocks were additionally sectioned and submitted to Fontana–Masson histochemical staining to assess melanin-associated pigmentation in the remaining fungal structures.

Molecular analysis was performed using formalin-fixed paraffin-embedded (FFPE) pulmonary tissue. DNA extraction was attempted from two 9-µm sections using three commercial extraction systems according to the manufacturers’ instructions: ReliaPrep™ FFPE gDNA Miniprep System (Promega, Madison, WI, USA), Quick-DNA/RNA Viral MagBead Kit (Zymo Research, Irvine, CA, USA), and GeneJET FFPE DNA Purification Kit (Thermo Fisher Scientific, Waltham, MA, USA).

To assess DNA integrity and amplifiability, a eukaryotic endogenous 18S rRNA gene fragment (252 bp) was targeted. Subsequently, two pan-fungal conventional PCR assays were performed targeting the internal transcribed spacer 2 (ITS2) region and the D1/D2 domain of the 28S rRNA gene. Positive and negative controls were included in all reactions. PCR products were analyzed by electrophoresis on 2% agarose gel stained with GelRed™ (Merck, Darmstadt, Germany).

## 3. Results

At necropsy, lesions were observed in multiple organs. The subscapular, inguinal, and abdominal aortic chain lymph nodes were enlarged and edematous, with a grayish-green coloration and multifocal blackened areas on cut surface.

In the abdominal cavity, a moderate amount of free blood-tinged fluid was observed, along with large blood clots. The liver was severely and diffusely congested, with moderate to severe steatosis, as evidenced by its pale coloration and increased size. A large thromboembolic mass was identified in the caudal vena cava, associated with vascular rupture and hemorrhage, which explained the presence of clots within the abdominal cavity.

**Figure 1:**
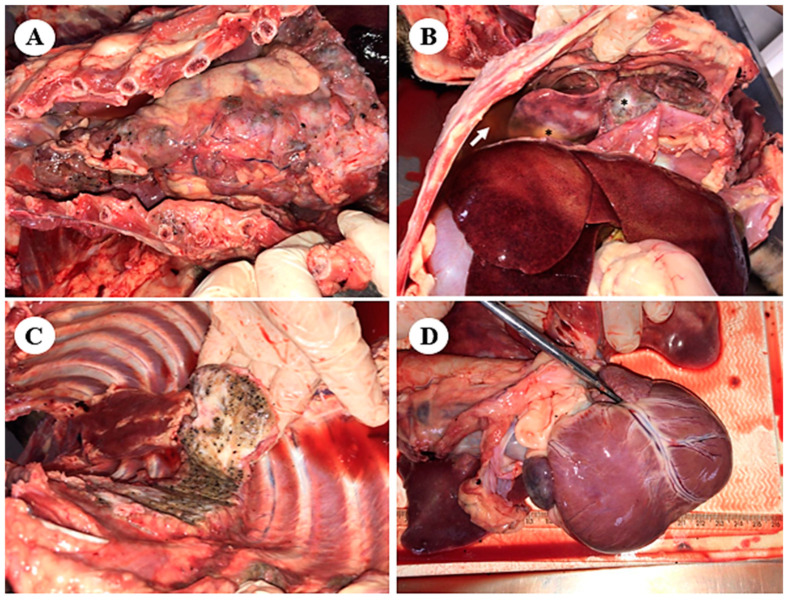
Phaeohyphomycosis in a dog. (**A**) Thoracic cavity opened by sternal ablation, showing severe pulmonary involvement characterized by multifocal to coalescing expansive nodular lesions, whitish in color with multifocal blackened areas, consistent with fungal colonies. (**B**) Opening of the thoracic cavity revealing severe hydrothorax (white arrow) and infiltrative whitish areas on the pulmonary pleura (*). (**C**) Costal pleura with a sectioned adhesion, showing a whitish mass with multifocal blackened areas, typical of dematiaceous fungal infection. (**D**) Heart showing severe right ventricular dilation, evidenced by bulging of the ventricular wall.

In the thoracic cavity, a large amount of free, non-coagulated yellowish-green fluid was present, accompanied by multiple extensive adhesions between the pulmonary lobes, pleura, and diaphragm. On cut section, these adhesions exhibited a solid appearance with a whitish-brown coloration and multifocal blackened areas (Figure 1A). The pulmonary lobes were severely affected, irregular, and displayed extensive multifocal to coalescing infiltrative nodular formations with similar characteristics to the adhesions, in addition to extensive areas of necrosis. The pulmonary pleura showed whitish areas with an expansive and infiltrative appearance (Figure 1B,C). The heart exhibited moderate to severe right ventricular dilation without myocardial wall thickening (Figure 1D).

**Figure 2:**
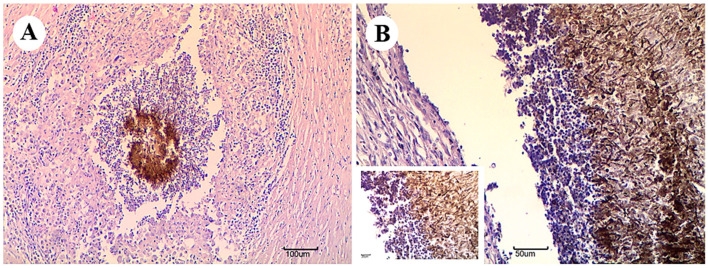
Diagnostic histopathology of phaeohyphomycosis in a dog. (**A**) Dematiaceous fungal colony within a granulomatous lesion in the lung. Note the brownish pigmentation resulting from melanin deposition in the fungal cell wall. Hematoxylin and eosin (H&E), 10×. (**B**) Colony of the same fungus observed in the lung, also present within the costal pleural adhesion mass. Note the inflammatory cells surrounding the fungal elements and the presence of epithelioid macrophages and fibroblasts composing the granuloma. Inset: pigmented, septate hyphae with branching angles <90° and rounded conidia at the extremities and free within the lesion. H&E, 20×.

Histologically, the pulmonary nodular lesions, pleural adhesions, and affected lymph nodes exhibited granulomas characterized by inflammatory infiltrates permeating the connective tissue, composed of macrophages, epithelioid macrophages, multinucleated giant cells, lymphocytes, and neutrophils. These inflammatory cells surrounded septate, dichotomously branching hyphae at acute angles, with parallel walls and transverse septa, presenting rounded terminal and free structures (conidia) and pigmentation ranging from brown to dark brown due to melanin deposition (Figure 2A,B).

Molecular analysis was performed using FFPE pulmonary tissue. No amplification was obtained for the endogenous 18S rRNA control gene, indicating marked nucleic acid degradation in the archived samples. Likewise, pan-fungal PCR assays targeting the ITS2 region and the D1/D2 domain of the 28S rRNA gene yielded no detectable amplicons. Based on the necropsy, histopathological, and histochemical findings, the case was diagnosed as pulmonary phaeohyphomycosis.

**Figure 3:**
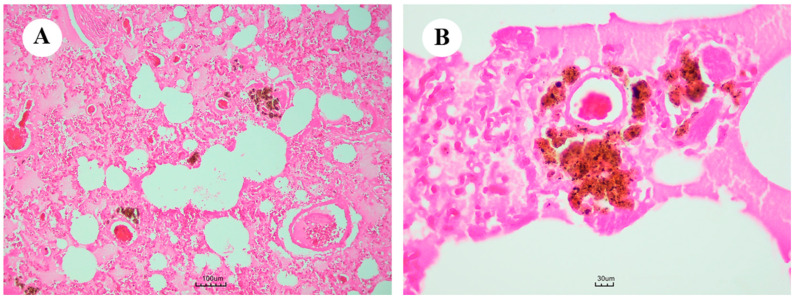
Fontana–Masson staining of residual pulmonary tissue from the dog. (**A**) Low-magnification view showing sparse pigmented fungal structures scattered within the pulmonary parenchyma. Fontana–Masson, 10×. (**B**) Higher magnification demonstrating dark brown to black positively stained fungal elements, consistent with melanin deposition in fungal cell walls. Fontana–Masson, 40×.

Additional histochemical evaluation using Fontana–Masson staining was performed on residual archived pulmonary paraffin blocks available during the revision process. Although the remaining material contained only sparse fungal elements, positively stained dark brown to black pigmented structures were observed within the pulmonary tissue, supporting the presence of melanin within fungal cell walls and reinforcing the diagnosis of infection caused by dematiaceous fungi (Figure 3).

## 4. Discussion

In the present study, an eight-year-old mixed-breed male dog was evaluated. Previous reports indicate that phaeohyphomycosis is more commonly described in felines [2]; however, cases have also been reported in dogs [1,4,7,8,9,10,11,12,13] and horses [5]. In dogs, the systemic form is the most frequently reported presentation, and no cases describing phaeohyphomycosis restricted exclusively to the thoracic cavity were found in the literature, as observed in the present case.

Regarding age, there are reports of canine cases involving animals of similar age to the one described here [4], as well as studies documenting affected dogs ranging from two to nine years of age and older cats, typically over eight years old [2].

Although the dog in this report was mixed-breed, cases have been described in German Shepherds [1,13], Siberian Huskies [11], Boxers [12], Fila Brasileiro dogs [7], and Cocker Spaniels [4]. As these are individual case reports, no clear breed or sex predisposition is currently recognized, likely reflecting the saprophytic and cosmopolitan nature of dematiaceous fungi [2].

Phaeohyphomycosis can affect both immunocompromised and immunocompetent individuals, with a higher prevalence in the former [3]. In the present case, the patient exhibited only lymphopenia, while other laboratory parameters remained within normal limits, similar to findings reported in previous studies [12,13]. One proposed explanation for infection in immunocompetent hosts is the role of fungal melanin in counteracting oxidative defense mechanisms of the host [20,21,22], thereby facilitating fungal survival and proliferation.

Although a definitive clinical diagnosis was not established ante mortem, the clinical signs observed in this case were consistent with those reported in the literature, including lethargy and weight loss [23], abdominal distension, dehydration, and multifocal lymphadenomegaly [4,24]. Some authors have described jaundice associated with hepatic involvement [4]; however, this finding was not observed in the present case.

Animals developing cutaneous lesions may exhibit alopecia, ulceration [12], and functional impairments such as lameness [11], local deformities, or even deep tissue involvement [13], depending on lesion location. In the present case, although cutaneous lesions were noted, laboratory findings did not support a fungal etiology, thereby excluding a cutaneous origin of infection.

Based on the severity and distribution of pulmonary and pleural lesions, a pulmonary origin of the mycotic infection is suggested, with subsequent spread to the pleura and lymph nodes, without direct involvement of the central nervous system, liver, or kidneys, as described in other reports [1,4]. Although the precise route of infection could not be determined, inhalation of fungal propagules is considered a plausible mechanism [3], particularly given that the infection was confined to the thoracic cavity.

The necropsy findings were consistent with those described in previous studies, regardless of the affected organ, including whitish areas covering serosal surfaces and extending into the parenchyma [1,7,23]. Nodular lesions have also been reported in affected tissues [4], as observed in this case, with severity varying according to tissue involvement and disease progression.

Secondary cardiomyopathy associated with pulmonary hypertension has been described as a consequence of increased pulmonary vascular resistance [25,26]. In the present case, chronic infectious pulmonary disease characterized by multiple granulomas and extensive necrosis likely impaired pulmonary blood flow, resulting in increased vascular resistance. This process may have led to right ventricular volume overload and subsequent dilation [27], as well as congestion of the caudal vena cava, contributing to the unfavorable outcome.

Histopathological findings were also consistent with previous descriptions [1,5,11,12], with granulomatous lesions containing pigmented fungal elements. Although molecular identification at the species level is desirable, a reliable diagnosis of phaeohyphomycosis can be established based on clinicopathological correlation and histopathological findings, particularly when pigmented hyphae compatible with dematiaceous fungi are observed within granulomatous inflammation. Septate, branching hyphae with brown to dark-brown pigmentation due to melanin deposition are characteristic features widely recognized as diagnostic criteria in both human and veterinary cases [2,3,6].

Although dichotomous branching at acute angles is classically described in invasive aspergillosis [28,29], several morphological characteristics observed in this case support infection by dematiaceous fungi. The fungal elements exhibited brown to dark-brown pigmentation compatible with melanin deposition within the cell wall, a hallmark feature of melanized fungi [3,6,22]. In addition, the presence of pigmented hyphae associated with granulomatous inflammation and conidial structures within the lesions supports the diagnosis of phaeohyphomycosis rather than hyalohyphomycosis caused by non-pigmented molds such as Aspergillus [2,3,30]. These features are recognized as important histopathological criteria for distinguishing infections caused by dematiaceous fungi in tissue sections [3,30].

Fontana–Masson staining performed during manuscript revision provided additional support for the diagnosis by demonstrating melanin-associated pigmentation in residual fungal structures present in archived pulmonary tissue. Although only limited material remained available and the most representative nodular lesions had been previously exhausted for molecular testing, the positive staining pattern was consistent with melanized fungi and further supported the diagnosis of phaeohyphomycosis.

Several dematiaceous fungi have been reported to cause systemic infections in dogs, including *Cladophialophora bantiana*, *Ochroconis gallopavum*, and *Curvularia* species [8,10,11,12]. These agents are frequently associated with disseminated, visceral, or central nervous system involvement in canine cases [8,10,12]. Although the specific etiological agent could not be identified in the present case, the clinicopathological findings are consistent with infections caused by these opportunistic melanized fungi, which have been previously reported to induce granulomatous lesions and, in some cases, vascular invasion in affected tissues [2,3,8,10].

The absence of amplification of the endogenous 18S control gene strongly suggests extensive nucleic acid degradation in the FFPE samples, which likely explains the failure of the pan-fungal PCR assays. DNA fragmentation is a well-recognized limitation of archived formalin-fixed tissues and may be further exacerbated by prolonged fixation, storage conditions, and the presence of inhibitory pigments such as melanin.

Previous studies indicate that in many cases of phaeohyphomycosis, particularly those diagnosed post mortem, etiological identification remains limited to morphological or genus-level classification due to lack of fresh samples for mycological culture or molecular amplification failure [3,31]. Although fungal culture is considered the gold standard for etiological diagnosis, it may not be feasible when samples are not collected or preserved appropriately during necropsy, or when fungal growth is slow or contaminated [30].

In this context, the present report highlights the importance of systematic sample collection during necropsy, including preservation of tissues in formalin, refrigeration, and freezing, to maximize diagnostic yield in opportunistic fungal infections. Nevertheless, even in the absence of molecular confirmation at the species level, the histopathological findings observed here are sufficient to support a diagnosis consistent with phaeohyphomycosis, as widely accepted in the specialized literature.

Fungal structures may also be observed within multinucleated giant cells and inside blood vessels, often associated with fibrin thrombus formation [32]. This finding suggests that intravascular fungal presence, combined with an intense inflammatory response, may have contributed to the development of caudal vena cava thromboembolism observed in this case, ultimately leading to vascular rupture due to hemodynamic overload and weakening of the vessel wall.

Venous thrombosis results from the interaction of multiple pathogenic factors, including endothelial injury, blood stasis, and hypercoagulable states, and is commonly observed in systemic inflammatory processes [33]. In dogs, spontaneous thrombosis involving the vena cava is uncommon but has been associated with conditions such as sepsis, neoplasia, immune-mediated diseases, protein-losing nephropathies, cardiac disease, and fungal infections, as observed in the present report [34].

In this case, severe and diffuse pulmonary inflammation likely played a central role in vascular pathophysiology by promoting systemic release of inflammatory mediators and toxins capable of impairing endothelial function and triggering vaso-occlusive pulmonary hypertension [35,36]. Consequently, increased venous return pressure, particularly affecting the caudal vena cava, favored thrombus formation and subsequent hemodynamic complications.

According to Ferreiro [2], the prognosis of phaeohyphomycosis ranges from guarded to poor. In the present case, severe pulmonary and pleural involvement, combined with cardiac alterations, led to progressive clinical deterioration. Due to the lack of a clinical diagnosis, appropriate treatment could not be instituted, ultimately resulting in patient death.

Treatment of phaeohyphomycosis represents a significant clinical challenge in both human and veterinary medicine, owing to the opportunistic behavior of dematiaceous fungi, variable clinical presentations, and frequent delays in diagnosis. Therapeutic approaches generally include systemic antifungal agents, alone or in combination with surgical resection of localized lesions when feasible [3,30], which was unlikely in the present case due to disease dissemination.

Among systemic antifungals, azoles—particularly itraconazole, voriconazole, and posaconazole—are most frequently used in the treatment of infections caused by dematiaceous fungi, demonstrating in vitro and in vivo activity against several causative agents of phaeohyphomycosis [30,37]. However, therapeutic response is variable and depends on factors such as extent of infection, host immune status, and lesion location, with disseminated forms associated with a guarded prognosis [37].

In veterinary medicine, successful therapeutic outcomes are more commonly reported in cases of localized cutaneous or subcutaneous infections diagnosed early, whereas systemic or pulmonary forms often progress unfavorably despite aggressive antifungal therapy [2,11]. In the present case, the absence of ante mortem etiological diagnosis and rapid disease progression precluded initiation of targeted antifungal treatment, likely contributing to the fatal outcome.

Therefore, this report emphasizes the importance of early recognition of opportunistic fungal infections in the differential diagnosis of chronic or progressive respiratory diseases in dogs, particularly when associated with nonspecific systemic signs. Early clinical suspicion may allow appropriate sample collection for culture and molecular identification, as well as timely initiation of antifungal therapy—factors considered critical for improving prognosis in cases of phaeohyphomycosis.

The routes of infection involved in phaeohyphomycosis remain incompletely understood; however, traumatic inoculation and inhalation of conidia are considered the primary mechanisms of entry for dematiaceous fungi. These organisms are widely distributed saprobes in the environment, particularly in soil, decaying vegetation, and organic debris, facilitating continuous exposure of animals and humans [3,6].

Although most veterinary reports describe cutaneous or subcutaneous infections associated with direct inoculation, the respiratory route has been increasingly recognized as an important mechanism in systemic or disseminated phaeohyphomycosis, particularly when the lung represents the primary site of infection [11,31]. Inhalation of fungal propagules may result in initial pulmonary colonization, followed by hematogenous dissemination to other organs, especially in the presence of intense inflammation or compromised alveolocapillary barrier integrity.

In the present case, the distribution and severity of pulmonary lesions, combined with the absence of cutaneous lesions suggestive of traumatic inoculation, support the hypothesis of inhalation as the route of infection, with the lung acting as the primary focus of disease. The intense inflammatory response observed in the pulmonary parenchyma may have facilitated vascular invasion by the fungal agent, contributing to systemic dissemination and thromboembolic complications.

Several predisposing factors have been associated with the development of phaeohyphomycosis, including immunosuppression, chronic disease, prolonged glucocorticoid therapy, neoplasia, and systemic inflammatory conditions [29,30]. Nevertheless, cases have also been reported in apparently immunocompetent hosts, suggesting that the virulence of dematiaceous fungi, together with the protective role of melanin against host defense mechanisms, plays a significant role in disease pathogenesis [6].

Thus, even in the absence of clearly identifiable predisposing factors, continuous environmental exposure and the probable inhalation route should be considered in the differential diagnosis of chronic or progressive respiratory conditions in dogs, particularly when accompanied by unfavorable clinical evolution and histopathological findings suggestive of opportunistic fungal infection.

Reports of phaeohyphomycosis in dogs remain relatively scarce, with most cases described as localized cutaneous or subcutaneous infections frequently associated with traumatic inoculation [2,11]. Cases involving systemic or visceral involvement are less common and generally associated with multisystemic lesions and poor clinical outcomes.

Compared with previously published reports, the present case is distinguished by the probable pulmonary origin of infection, associated with intense local inflammatory response, vascular invasion by the fungal agent, and development of large-vessel thrombosis culminating in caudal vena cava thromboembolism. Although hematogenous dissemination is recognized in dematiaceous fungal infections, pulmonary involvement as a primary focus in dogs remains poorly documented, being more frequently reported in human cases or experimental studies [3,31].

Furthermore, the association between pulmonary phaeohyphomycosis and severe vascular complications, such as large-vessel thrombosis and pulmonary hypertension, is rarely reported in veterinary medicine, underscoring the unusual nature of this case. These findings expand the spectrum of clinical manifestations attributed to dematiaceous fungal infections in dogs and highlight the need to consider phaeohyphomycosis as a differential diagnosis in cases of chronic or progressive respiratory disease accompanied by systemic signs and thromboembolic events.

In conclusion, this report contributes to the veterinary literature by documenting an atypical and fatal presentation of canine phaeohyphomycosis, characterized by a probable inhalation route of infection, primary pulmonary involvement, and severe vascular complications, providing relevant insights for the recognition, diagnosis, and clinical management of similar cases.

## 5. Conclusions

This case report describes an uncommon and fatal presentation of phaeohyphomycosis in a dog, characterized by a probable inhalation route of infection, primary pulmonary involvement, and severe vascular complications, including large-vessel thrombosis. Although molecular identification of the etiological agent was not achieved, the histopathological findings, together with the clinicopathological correlation, were sufficient to support a diagnosis consistent with phaeohyphomycosis, as widely accepted in the scientific literature.

This case highlights the diagnostic and therapeutic challenges associated with infections caused by dematiaceous fungi in veterinary medicine, particularly in the context of progressive respiratory disease accompanied by nonspecific systemic manifestations. Furthermore, it underscores the importance of early clinical suspicion, appropriate sample collection, and integration of clinical, pathological, and laboratory data for the recognition of these opportunistic infections.

Finally, this report expands the spectrum of clinical manifestations attributed to phaeohyphomycosis in dogs and emphasizes the need to include this disease in the differential diagnosis of chronic or severe respiratory disorders, thereby contributing to improved diagnostic accuracy, clinical management, and understanding of the pathogenesis of these infections in veterinary practice.

## Data Availability

The original contributions presented in this study are included in the article. Further inquiries can be directed to the corresponding author.

## Abbreviations

The following abbreviations are used in this manuscript:

ALT	Alanine Aminotransferase
ALP	Alkaline Phosphatase
APC	Article Processing Charge
A-FAST	Abdominal Focused Assessment with Sonography for Trauma
bpm	beats per minute
CEUA	Ethics Committee on Animal Use
H&E	Hematoxylin and Eosin
IU	International Units
MCHC	Mean Corpuscular Hemoglobin Concentration
MCV	Mean Corpuscular Volume
PCR	Polymerase Chain Reaction
UFJ	Federal University of Jataí
UPC	Urine Protein-to-Creatinine Ratio
